# Autophagy promotes fibrosis and apoptosis in the peritoneum during long‐term peritoneal dialysis

**DOI:** 10.1111/jcmm.13393

**Published:** 2017-10-27

**Authors:** Jingjing Wu, Changying Xing, Li Zhang, Huijuan Mao, Xuguan Chen, Mingxing Liang, Fang Wang, Haibin Ren, Hongqing Cui, Aiqin Jiang, Zibin Wang, Meijuan Zou, Yong Ji

**Affiliations:** ^1^ Department of Pharmacology School of Basic Medical Sciences Nanjing Medical University Nanjing Jiangsu China; ^2^ Department of Nephrology First Affiliated Hospital Nanjing Medical University Nanjing Jiangsu China; ^3^ Key Laboratory of Cardiovascular and Cerebrovascular Medicine School of Pharmacy Nanjing Medical University Nanjing Jiangsu China; ^4^ Department of Cardiology First Affiliated Hospital of Nanjing Medical University Nanjing China; ^5^ Medical School of Nanjing University Nanjing China; ^6^ Analytical & Testing Center Nanjing Medical University Nanjing Jiangsu China

**Keywords:** high‐glucose peritoneal dialysis solution, Beclin 1‐dependent autophagy, fibrosis, apoptosis, human peritoneal mesothelial cells

## Abstract

Long‐term peritoneal dialysis is accompanied by functional and histopathological alterations in the peritoneal membrane. In the long process of peritoneal dialysis, high‐glucose peritoneal dialysis solution (HGPDS) will aggravate the peritoneal fibrosis, leading to decreased effectiveness of peritoneal dialysis and ultrafiltration failure. In this study, we found that the coincidence of elevated TGF‐β1 expression, autophagy, apoptosis and fibrosis in peritoneal membrane from patients with peritoneal dialysis. The peritoneal membranes from patients were performed with immunocytochemistry and transmission electron microscopy. Human peritoneal mesothelial cells were treated with 1.5%, 2.5% and 4.25% HGPDS for 24 hrs; Human peritoneal mesothelial cells pre‐treated with TGF‐β1 (10 ng/ml) or transfected with siRNA Beclin1 were treated with 4.25% HGPDS or vehicle for 24 hrs. We further detected the production of TGF‐β1, activation of TGF‐β1/Smad2/3 signalling, induction of autophagy, EMT, fibrosis and apoptosis. We also explored whether autophagy inhibition by siRNA targeting Beclin 1 reduces EMT, fibrosis and apoptosis in human peritoneal mesothelial cells. HGPDS increased TGF‐β1 production, activated TGF‐β1/Smad2/3 signalling and induced autophagy, fibrosis and apoptosis hallmarks in human peritoneal mesothelial cells; HGPDS‐induced Beclin 1‐dependent autophagy in human peritoneal mesothelial cells; Autophagy inhibition by siRNA Beclin 1 reduced EMT, fibrosis and apoptosis in human peritoneal mesothelial cells. Taken all together, these studies are expected to open a new avenue in the understanding of peritoneal fibrosis, which may guide us to explore the compounds targeting autophagy and achieve the therapeutic improvement of PD.

## Introduction

Peritoneal dialysis (PD), using the peritoneal membrane (PM) as semipermeable barrier for the exchange of toxic substances and water, is an effective alternative means of renal replacement therapy for patients with end‐stage renal failure (ESRF). PD is currently used by about 11% of dialysis patients with ESRF 1, 2, 3. The long‐term exposure to hyperosmotic, hyperglycaemic and low pH of dialysis solutions is accompanied by injury of PM with functional and histopathological alterations in the peritoneum, which leads to the ultrafiltration failure in patients with PD 2. Human peritoneal mesothelial cells (HPMCs) are the main component of PM structure. Thus, the cellular function level of HPMCs plays a pivotal role in dialysis adequacy 4, 5. Convincing evidence has highlighted the harmful nature of conventional HGPDS on the structural, functional, and morphologic properties of HPMCs, attributed in part to a series of cytological and pathological consequences including activation of intracellular signal transduction pathway 6, 7.

The production of TGF‐β1, stimulated by glucose, acid pH and infections, is the dominant extracellular matrix (ECM) and collagen‐producing factor during organ fibrosis 8. Predominantly, the excessive amount of TGF‐β1 expression is associated with worse PD outcome 9, 10. TGF‐β1 signal *via* heterodimeric serine/threonine kinase transmembrane receptor complexes exerts its biological effects by activating the phosphorylation of Smad2 and Smad3, which are negatively regulated by an inhibitory Smad7 11. The binding of the ligand to its primary receptor (TGF‐β receptor type II, TGF‐β RII) allows the recruitment, transphosphorylation and activation of the signalling receptor (TGF‐β receptor type I, TGF‐β R I). TGF‐β R I, subsequently exhibits its serine‐threonine kinase activity of phosphorylating Smad2 and Smad3 9, 12. Smad3 signalling triggered TGF‐β1‐induced EMT and fibrosis that has been demonstrated that Smad3 knockout mice can be protected from peritoneal fibrosis (PF) with loss of collagen accumulation 13.

Autophagy works as a conserved proteolytic mechanism, through which cytoplasmic components are sequestered into specialized double‐membrane‐bound autophagosomes and degraded by lysosomes and for subsequently recycling 14. The clearance of damaged organelles and protein by autophagy plays an important role in maintaining cellular homeostasis 15. The autophagic sequestration progression contains two series of multiprotein components. The first complex includes a protein kinase Apg1, not yet characterized in mammals 16. The second complex contains PI3K (phosphoinositide 3‐kinase) of class III, its adapter p150 and Beclin1 (mammalian Vps34, Vps15 and Apg6, respectively) 17. The class III phosphoinositide 3‐kinase‐Beclin1 complex has been reported to mediate the localization of autophagy proteins to autophagic vesicles 18, 19.

Autophagy is reported to contribute to apoptosis as type II programmed cell death *via* autodigestive cellular progression or extracellular stimulation 20, 21. Autophagy is previously observed as a regulator of TGF‐β1‐induced fibrogenesis in primary human atrial myofibroblasts 22. However, there is little evidence linking autophagy with injury of PM during the functional and histopathological alterations such as apoptosis and fibrogenesis in HPMCs. In this study, we illustrated the Beclin 1‐dependent autophagy during fibrosis and apoptosis induced by HGPDS in HPMCs and demonstrate that a new avenue that autophagy inhibition can weaken the fibrosis and apoptosis, indicating that targeting autophagy may achieve the therapeutic improvement of PD.

## Materials and methods

### Reagents and antibodies

The 1.5%, 2.5% and 4.25% PD solution was purchased from Baxter USA (Glenview, IL, USA). The recombinant human TGF‐β1 (#AF‐100‐21C; PeproTech, Rocky Hill, CT, USA) was dissolved in triple‐distilled water at the concentration 100 μg/ml and stored at −80°C. A stock solution was diluted to the concentration 10 ng/ml with medium as the working concentration.

Primary antibodies to MAP‐LC3 (12741), TGF‐β1 (3711), Smad2/3 (8685), phospho‐Smad2 (3104), phospho‐Smad3 (9520) and Beclin 1 (4122) were purchased from Cell Signaling Technology, Inc. (Beverly, MA, USA). Primary antibodies to E‐cadherin (1702‐1), N‐cadherin (2447‐1) and Vimentin (2862‐1) were obtained from Epitomics Inc. (Burlingame, CA, USA). Primary antibodies to TGF‐β R I (55391‐1‐AP), Snail (26183‐1‐AP), Twist1 (25465‐1‐AP), Slug (12129‐1‐AP), Caspase‐8 (13423‐1‐AP), Fibronectin (15613‐1‐AP), p62/SQSTM1 (18420‐1‐AP), collagen 1α2 (14695‐1‐AP) were purchased from Proteintech Technology, Inc. (Wuhan, China). Primary antibodies to TGF‐β R II (sc‐400) were purchased from Santa Cruz Technology, Ltd. (Santa Cruz, CA, USA). Primary antibodies to PARP (70001), Bax (1030), Mcl‐1 (6490) and Bcl‐2 (1031) were obtained from Bioworld Technology Inc. (Minneapolis, MN, USA). The GAPDH antibody was purchased from Boster (Wuhan, China). The secondary antibodies horseradish peroxidase (HRP)‐conjugated labelled goat antimouse IgG (H+L) (111‐035‐003), peroxidase‐conjugated labelled goat anti‐rabbit IgG (H+L) (111‐035‐003), fluorescein FITC‐conjugated affinipure goat anti‐rabbit IgG (H+L) (111‐095‐003) were obtained from Jackson Immuno Research Laboratories, Inc. (West Grove, PA, USA).

### Cell culture and human PM samples

Human peritoneal mesothelial cells, obtained from ATCC cell bank (MeT‐5A, number CRL‐9444), were maintained in Dulbecco's modified eagle medium (DMEM; Gibco Inc., Grand Island, NY, USA) supplemented with 10% fetal bovine serum, 100 U/ml penicillin and streptomycin. Cells were incubated in a humidified atmosphere at 37°C in 5% CO_2_, and the culture medium was changed every 2–4 days. Cells were liberated and subcultured with trypsin‐EDTA (ethylene diamine tetraacetic acid; Gibco Inc., Grand Island, NY). Only cells from 3 to 8 generations were used for the experiments.

The four parietal peritoneal biopsy samples from four cases patients with catheterization or extubation for PD at the First Affiliated Hospital of Nanjing Medical University were collected from the side opposite the catheter installation or extraction. Table 1 is the information about patients from catheterization or extubation for PD. The PM tissues were randomly selected for immunohistochemistry (IHC) assay and transmission electron microscopy. The experiments were undertaken with the understanding and written consent of each subject. The study methodologies conformed to the standards set by the Declaration of Helsinki. The study methodologies were approved by Ethics Committee of First Affiliated Hospital, Nanjing Medical University (Ethical No: 2017‐SRFA‐009).

**Table 1:** Information about patients from catheterization or extubation for peritoneal dialysis

Patients	Gender	Age (years)	Protopathy	Time of dialysis (years)	Operation
1	Male	25	CG	0	Catheterization
2	Male	47	CG	0	Catheterization
3	Male	35	CG	3	Extubation
4	Male	45	CG	5	Extubation

CG: Chronic Glomerulonephritis.

### Immunocytochemistry

After being fixed for 24 hrs in 4% paraformaldehyde at 4°C, tissues from PM were embedded in paraffin and serially sectioned in 5 μm sections. PM thickness was determined using light microscopy (Leica CTR6000 with a Leica Microsystems LAS‐AF6000, Leica Biosystems, Inc., Buffalo Grove, IL, USA). Sections were stained with TGF‐β1, MAP‐LC3, Beclin 1, N‐cadherin antibodies (dilution 1:50) overnight at 4°C. After the tissue slides were washed, they were incubated with anti‐rabbit IgG HRP secondary antibody for 10 min. The slides were stained with 3, 3′‐diaminobenzidine (DAB; Vector Laboratories, Burlingame, CA, USA), counterstained with haematoxylin (Vector Laboratories, Burlingame, CA, USA), dehydrated, treated with xylene and mounted. All slides were examined, and microscope photographs were obtained using an Olympus BX41 microscope (Olympus America, Melville, NY, USA).

### Transmission electron microscopy

The fresh peritoneal biopsy samples including each patient before and with PD at were collected from the side opposite the catheter installation or extraction. For experiment *in vitro*, HPMCs were treated with 4.25% HGPDS for 24 hrs. Immediately, fresh peritoneal biopsy samples or cells were fixed with 1% glutaraldehyde and post‐fixed with 2% osmium tetroxide. Then, the cell pellets or sections are embedded in Epon resin. Representative areas are chosen for ultrathin sectioning and viewed using a FEI Tecnai G2 Spirit Bio TWIN transmission electron microscope (FEI Co., Eindhoven, the Netherlands) at an acceleration voltage of 120 kV.

### Transfection of siRNA interference

For siRNA transfection, we took Lipofectamine™ 3000 kit (Invitrogen, Carlsbad, CA, USA) according to the manufacturer's instructions. Briefly, cells were trypsinized and seeded in 6‐well plates at a density of 5000 cells/well for 18 hrs. Cells that reached 50% confluence were transfected with serum‐free DMEM medium containing 100 nM siRNA Control, siRNA Beclin 1 for 6 hrs, followed by recovery in medium containing serum and 4.25% HGPDS treatment as necessary. The human Beclin‐1 siRNA oligonucleotides (Sequence 1: sense 5′‐AAGAUUGAAGACACAGGAGGC‐3′, antisense 5′‐GCCUCCUGUGUCUUCAAUCUU‐3′; Sequence 2: sense 5′‐CAGUUUGGCACAAUCAAUA‐3′, antisense 5′‐UAUUGAUUGUGCCAAACUG‐3′) were synthesized by GenePharma, Inc. (Pudong, Shanghai). A universal negative control siRNA is used.

### Enzyme‐linked immunosorbent assay (ELISA)

The levels of TGF‐β1 from Control, 1.5%, 2.5% and 4.25% HGPDS‐treated peritoneal mesothelial cells were determined by ELISA it (R&D, system Inc., Minneapolis, MN, USA) Cells were treated with 1.5%, 2.5% and 4.25% HGPDS for 24 hrs. After that, cells were washed with PBS carefully and cultured in serum‐free DMEM medium. After another 24 hrs, the DMEM medium was collected and centrifuged (13400 g, 10 min.). The secretion of TGF‐β1 from this supernatant was determined by ELISA kit according to the manufacturer's instructions.

### Western blotting assay

Human peritoneal mesothelial cells were treated as mentioned above. Cells were washed twice with PBS and lysed in lysis buffer containing 50 mM Tris‐Cl [pH 7.4], 150 mM NaCl, 1% Triton X‐100, 1% deoxycholic phenylmethylsulfonyl fluoride, 1 mg/ml aprotinin, 5.0 mM sodium pyrophosphate, 1.0 g/ml leupeptin, 0.1 mM phenylmethylsulfonyl floride and 1 mM DTT. Cells were clarified by centrifugation (13 000 g, 15 min., 4°C). Bicinchoninic acid (BCA) kit with Varioskan multimode microplates spectrophotometer from Thermo Fisher Scientific Inc. (Waltham, MA, USA) was used to detect the concentration of protein in supernatants.

For sample loading, equal amounts of protein (20–30 μg) were separated by 10%, 12% or 15% sodium dodecyl sulphate polyacrylamide gel electrophoresis (SDS‐PAGE) and transferred onto the 0.22 μm polyvinylidene fluoride (PVDF) membranes (Millipore, Boston, MA, USA). The immune complexes were formed by incubation of proteins with primary antibodies overnight at 4°C followed by the appropriate HRP‐labelled secondary antibodies for 1 hr at 37°C. Detection of the immune blots was performed using the ECL plus Western blotting detection reagents (Bio‐Rad, Hercules, CA, USA) and the ChemiDoc XRS Plus luminescent image analyzer (Bio‐Rad) 23.

### Total RNA extraction, RT‐PCR for mRNA

Total RNA was extracted using TRIzol reagent (Invitrogen). Reverse transcription‐PCR was performed with M‐MLV (Promega, Madison, WI, USA) following standard protocols. For the TaqMan‐based real‐time reverse transcription–polymerase chain reaction (RT‐PCR) assays, ABI 7900 HT Sequence Detection system (Applied Biosystem, Foster City, CA) was used. For quantitative PCR of mRNA, the MMP‐2, MMP‐9, and GAPDH primers were purchased from Invitrogen (Life Technology). EzOmics SYBR qPCR kit was purchased from Biomics. Amplification procedure was 94°C for 5 min., followed by 30 cycles at 94°C for 30 sec., 61°C for 45 sec., finally 72°C for 10 min. The primers used were as follows: (*i*) human MMP‐2, forward primer: 5′‐GACAACGCCCCCATACCAG‐3′, and reverse primer: 5′‐CACTCGCCCCGTGTGTTAGT‐3′; (*ii*) human MMP‐9, forward primer: 5′‐ACGCAGACATCGTCATCCAGT‐3′, and reverse primer: 5′‐GGACCACAACTCGTCATCGTC‐3′; (*iii*) human GAPDH, forward primer: 5′‐AAGGTCGGAGTCACCGGATT‐3′, and reverse primer: 5′‐AAGGTCGGAGTCACCGGATT‐3′.

### Autophagy detection and flow cytometry

Autophagy induction of HPMCs was determined using SNLYSO autophagy detection kit (E0010; SNPT, Chengdu, China), according to the manufacturer's instructions. Cells were gently washed by PBS after treatment with indicated condition. Then 250 μl SNLYO sensor was added to incubated the cells for 20 hrs. The cells were digested with EDTA‐free trypsin and analysed at the density of 10^5^–10^6^/ml by BD FACSCalibur™ Flow Cytometry System (Franklin Lakes) and a computer station running Cell Quest software (BD Biosciences, Franklin Lakes, NJ, USA).

### Wound‐healing assay

Wound‐healing assay was applied to study directional cell migration induced by HGPDS treatment. The HPMCs seeded in a 6‐well plate for 18 hrs to reach 80% confluence. Subsequently, cell monolayers were wounded a cross using white sterile micropipette tips and washed twice with PBS to remove floating cells. Then, cells transfected with siRNA Beclin 1 or vehicle were treated with 4.25% HGPDS for 24, 48 and 72 hrs after wounding. Then the migrating cells into the denuded zone were assessed using an inverted microscope. Image magnification: 10×.

### Annexin V/PI staining

Apoptotic death was detected using Annexin V‐FITC Apoptosis Detectin Kit I (556547; BD Biosciences, Qume Drive, San Jose, CA, USA) according to the manufacturer's protocol. HPMCs pre‐treated with TGF‐β1 (10 ng/ml) or transfected with siRNA Beclin1 were treated with 4.25% HGPDS or vehicle for 24 hrs. 1 × 10^6^ cells were then harvested, washed with PBS and resuspended in 500 μl binding buffer (pH 7.5, 10 mM HEPES, 2.5 mM CaCl_2_, and 140 mM NaCl). Then 5 μl Annexin V‐FITC and 1 μl PI were added in the cell suspension and incubated avoiding light for 10 min. at room temperature. Finally, cells were analysed by BD FACSCalibur™ Flow Cytometry System (BD Biosciences, Franklin Lakes) and a computer station running Cell Quest software (BD Biosciences, Franklin Lakes).

### Statistical analyses

Data from three independent experiments were expressed as mean ± S.D. and statistically compared by one‐way anova with Dunnett's test and *post hoc* tests were undertaken using the SigmaPlot 10.0 software (Systat Software, Inc, San Jose, CA, USA). The details of each statistical analysis used were presented in the figure legends. Significance was indicated as *P < 0.05, #*P* < 0.05 and **P < 0.01, ##*P* < 0.01.

## Results

### Coincidence of elevated TGF‐β1 expression, autophagy, apoptosis and fibrosis in PM from patient with PD

**Figure 1:**
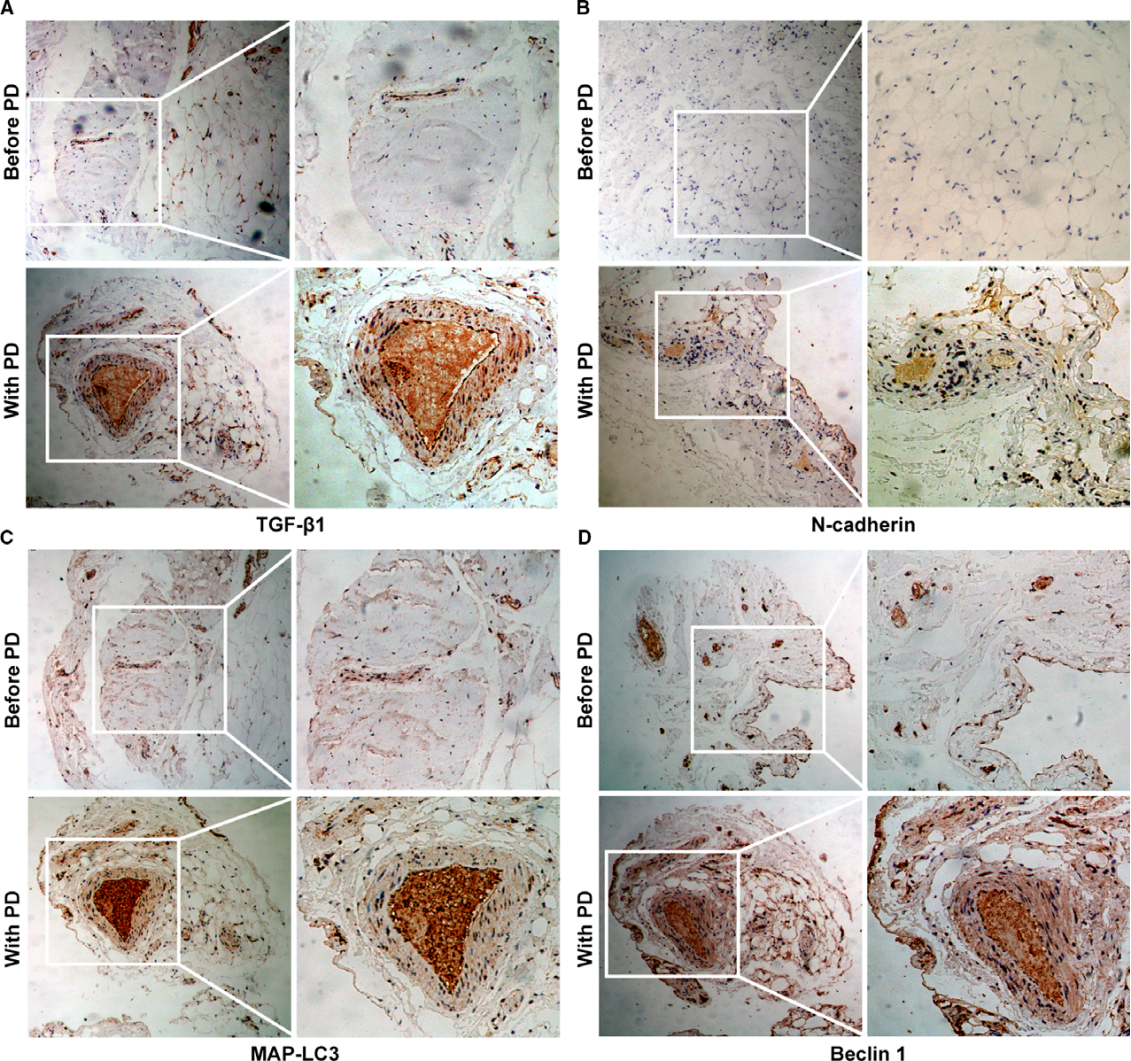
Expression of TGF‐β1, autophagy and EMT hallmarks in peritoneal membrane (PM) from patients before and with PD. (**A–D**) The PM tissues of each patient before and with PD were randomly selected for immunohistochemistry (IHC) assay. Representative photomicrographs of TGF‐β1, N‐cadherin, MAP‐LC3 and Beclin 1 expression as determined by IHC staining in peritoneum samples of patients before PD and with PD. Image magnification: 100× (left); 200× (right).

Long‐term exposure to dialysis solutions with hyperosmotic, hyperglycaemic and low pH and repeated episodes of peritonitis progressively triggered fibrosis of the peritoneum, which leads to the termination of dialysis 24. In addition, apoptosis in peritoneal mesothelial cells is considered to be another early mechanism of peritoneal injury 25. The production of TGF‐β1 in the peritoneum microenvironment is considered the major cause of changes of cell morphology and movement. The PMs of patients before PD and with PD were randomly selected for IHC assay. Representative photomicrographs of TGF‐β1 (Fig. 1A), N‐cadherin (Fig. 1B), MAP‐LC3 (Fig. 1C) and Beclin 1 (Fig. 1D) expressions were determined by IHC staining. The data presented here show that HGPDS‐induced up‐regulation of autophagy and EMT markers is associated with TGF‐β1 expression in PM from patient with PD (Fig. 1).

**Figure 2:**
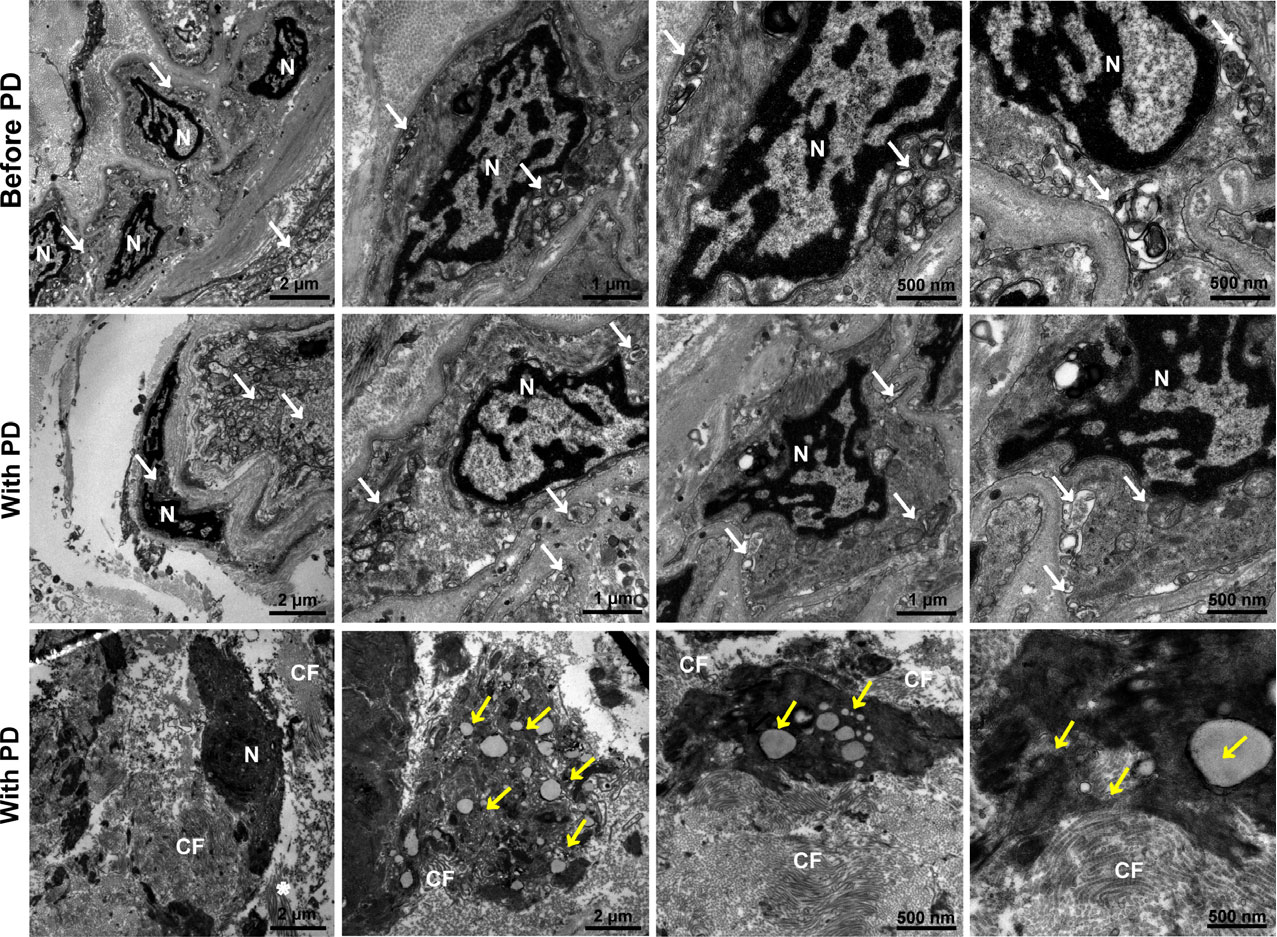
Characteristic morphology of autophagy, fibrosis and apoptosis of peritoneal membrane from patients before and with PD under transmission electron microscope. Transmission electron microscopy images of the peritoneum tissues of each patient before and with PD. Tissues were fixed immediately in 1% glutaraldehyde, post‐fixed in 2% osmium tetroxide and embedded in Epon resin. Representative areas were picked for ultrathin sectioning. Transmission electron microscopy is performed using a Tecnai G2 Spirit Bio TWIN (FEI Co.) at 120 kV. N: nuclear; CF: Collagen fibre; White arrows, autophagosomes; Yellow arrows, apoptotic empty vacuoles. Scale bars: 1 μm, 500 nm (right) and 2 μm (left).

Transmission electron microscopy analysis of PMs of patients before PD and with PD revealed increasing structures of autophagy, apoptosis and fibrosis. Figure 2 clearly shows characteristic morphology of cell substructure such as autophagosome, autophagolysosmes, typical collagen fibre and apoptotic empty vacuoles of PM from patients with PD under transmission electron microscope.

### HGPDS increased TGF‐β1 production, activated TGF‐β1/Smad2/3 signalling and induced autophagy, fibrosis and apoptosis hallmarks in HPMCs

**Figure 3:**
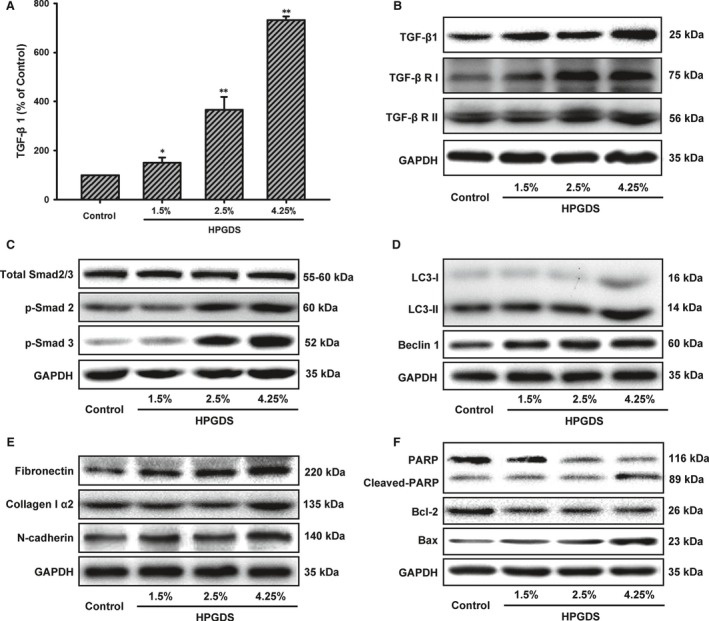
High‐glucose peritoneal dialysis solution (HGPDS) increased TGF‐β1 production, activated TGF‐β1/Smad2/3 signalling and induced autophagy, fibrosis and apoptosis hallmarks in human peritoneal mesothelial cells (HPMCs). (**A**) The levels of TGF‐β1 from Control, 1.5%, 2.5% and 4.25% HGPDS‐treated cells were determined by ELISA. Each bar represents the mean ± S.D. Statistical analysis was performed using Student's *t*‐test, *P < .05, **P < .01. (**B** and **C**) HGPDS induced expression of TGF‐β1 and activated TGF‐β1/Smad2/3 signalling in HPMCs. (**D**) Effect of HGPDS on MAP‐LC3 lipidation and Beclin 1 expression levels in cells is analysed by Western blotting. HPMCs were treated with 1.5%, 2.5%, and 4.25% HGPDS for 48 hrs. (**E** and **F**) HPMCs were treated with 1.5%, 2.5%, and 4.25% HGPDS for 48 hrs. Western blotting revealed that HGPDS induced expression of both fibrosis and apoptosis hallmarks. GAPDH was used as internal controls to ascertain equal loading.

We firstly examined HGPDS‐induced synthesis of TGF‐β1 in HPMCs. Cells were treated with 1.5%, 2.5% and 4.25% HGPDS for 24 hrs. After that, cells were washed with PBS carefully and cultured in serum‐free DMEM medium. After another 24 hrs, the DMEM medium was collected and centrifuged (12,000 r.p.m., 10 min.). The secretion of TGF‐β1 from this supernatant was determined by ELISA kit. As a result, the stimuli of HPMCs with HGPDS significantly increased the production of TGF‐β1 in HGPDS group as compared with the control group (Fig. 3A). To further identify TGF‐β1/Smad2/3 pathway might be involved in autophagy‐related apoptosis and fibrosis, Western blotting analysis was conducted. Figure 3B indicates that treatment of cells with 1.5%, 2.5% and 4.25% HGPDS for 24 hrs induced significant increase in TGF‐β1, TGF‐β Receptor I (TGF‐β RI) and TGF‐β Receptor II (TGF‐β RII) expression levels compared to untreated control treatment. As following, HGPDS increases Smad2 and Smad3 phosphorylation, indicating the activation of TGF‐β1/Smad2/3 pathway (Fig. 3C).

Our results then show that HGPDS (1.5%, 2.5% and 4.25%) induces significant increases in the synthesis of collagen type Iα2, fibronectin and N‐cadherin in the presence of LC3β II lipidation and Beclin 1 (Fig. 3D and E). We also detected the expression of apoptotic protein Bax, anti‐apoptotic proteins Bcl‐2 and PARP. Bax expression increased while expression Bcl‐2 decreased in cells treated with HGPDS. Thus, the ratio of Bax/Bcl‐2 increased in cells treated with HGPDS, which is crucial for the activation of mitochondrial apoptotic pathway (Fig. 3F). As shown in Figures 3F, PARP cleavage increased as concentration of HGPDS increased. These findings all together indicated that HGPDS increased TGF‐β1 production, activated TGF‐β1/Smad2/3 signalling and induced autophagy, fibrosis and apoptosis hallmarks in HPMCs.

### HGPDS‐induced Beclin 1‐dependent autophagy in HPMCs

**Figure 4:**
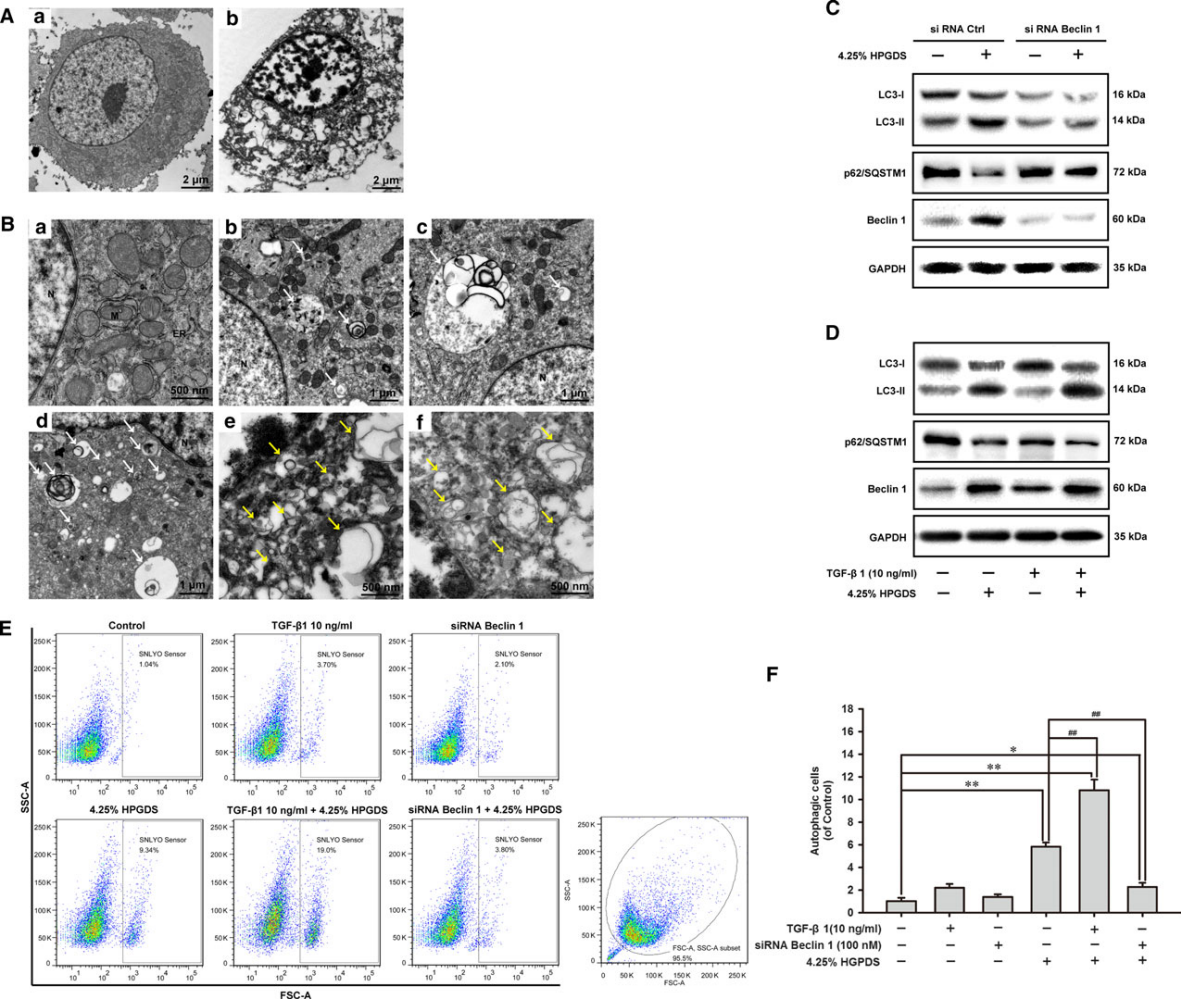
HPGDS induced Beclin 1‐dependent autophagy in human peritoneal mesothelial cells (HPMCs). (**A** and **B**) Transmission electron microscopy images of HPMCs following high‐glucose peritoneal dialysis solution (HGPDS) treatment. The HPMCs were treated with 4.25% HGPDS or vehicle for 24 hrs. Cells were then fixed immediately in 1% glutaraldehyde and post‐fixed in 2% osmium tetroxide. The cell pellets or sections were embedded in Epon resin. Representative areas were picked for ultrathin sectioning. Transmission electron microscopy is performed using a Tecnai G2 Spirit Bio TWIN (FEI Co.) at 120 kV. (**A**) The HPMCs were treated with vehicle as control (a) and 4.25% HGPDS for 24 hrs (b). Scale bars: 2 μm. (**B**) Cells treated with vehicle as a control with normal nuclear and organelles. N: nuclear; M: mitochondrion; ER: endoplasmic reticulum. Scale bars: 500 nm (a). Cells are treated with 4.25% HGPDS for 24 hrs. N: nuclear; White arrows, representative autophagic structures; Yellow arrows, degradative autophagic vacuoles. Scale bars: 1 μm (b, c and d), 500 nm (e and f). (**C**) Western blotting analysis shows the effect of 4.25% HPGDS treatment of cells transfected with siRNA Beclin 1 or siRNA Ctrl on MAP‐LC3 lipidation and p62/SQSTM1 expression. (**D**) Western blotting analysis shows the effect of 4.25% HPGDS treatment of cells cotreated with TGF‐β1 (10 ng/ml) on MAP‐LC3 lipidation and p62/SQSTM1 expression. (**E**) and (**F**) HPMCs pre‐treated with TGF‐β1 (10 ng/ml) or transfected with siRNA Beclin1 were treated with 4.25% HGPDS or vehicle for 24 hrs. Cells were then probed by SNLYSO sensor (an autolysosome florescent probe), and autophagic cells were analysed and quantitated by flowcytometer. Results from three independent experiments are shown as means ± S.D. **P < 0.01, *P < 0.05 *versus* untreated control group. ^##^ *P* < 0.01 *versus* 4.25% HGPDS‐treated group.

Transmission electron microscopy analysis of HPMCs treated with 4.25% HGPDS was applied to gain insight into the cell substructure morphological changes. As a gold standard to detect autophagosome, images from TEM revealed accumulation of autophagy‐related structures and apoptosis empty vacuole in 4.25% HGPDS‐treated cells compared to control cells (Fig. 4A). HPMCs treated with vehicle as a control are full of normal nuclear, organelles such as mitochondrion and endoplasmic reticulum (Fig. 4A [a]). Detailed images revealed the dynamic process of autophagy. The rough endoplasmic reticulum without ribosomes formed the double isolation membrane structures, which wrapped around degradation organelles, proteins and other components. The autophagosome fused with lysosomes to form the autolysosome, where the compartments were finally digested (Fig. 4B [b–f]).

Based on the above illustration in TEM images, we further explore whether Beclin 1 is involved in the autophagy induction by HGPDS. Western blotting analysis also showed that 4.25% HPGDS treatment of cells transfected with siRNA Beclin 1 rescued MAP‐LC3 lipidation as well as the degradation of p62/SQSTM1 compared with siRNA Ctrl (Fig. 4C). Conversely, TGF‐β1 (10 ng/ml) increased MAP‐LC3 lipidation and the degradation of p62/SQSTM1 in HPMCs (Fig. 4D). Western blotting analysis shows the effect of 4.25% HPGDS treatment of cells cotreated with TGF‐β1 (10 ng/ml) on MAP‐LC3 lipidation and p62/SQSTM1 expression. Further we investigated the effect of siRNA Beclin 1 and TGF‐β1 (10 ng/ml) on autophagy induced by 4.25% HPGDS using SNLYSO autophagy detection kit and flowcytometer. It was shown in Figure 4E and F, the autophagy induction of 4.25% HPGDS was significantly reduced by siRNA Beclin 1 (from 9.41 ± 0.56% to 3.63 ± 0.62%). While the autophagy induction of 4.25% HPGDS was increased by TGF‐β1 (10 ng/ml) (from 9.41 ± 0.56% to 17.43 ± 1.55%). Thus, HGPDS‐induced Beclin 1‐dependent autophagy in HPMCs.

### Autophagy inhibition reduced EMT and fibrosis in HPMCs

**Figure 5:**
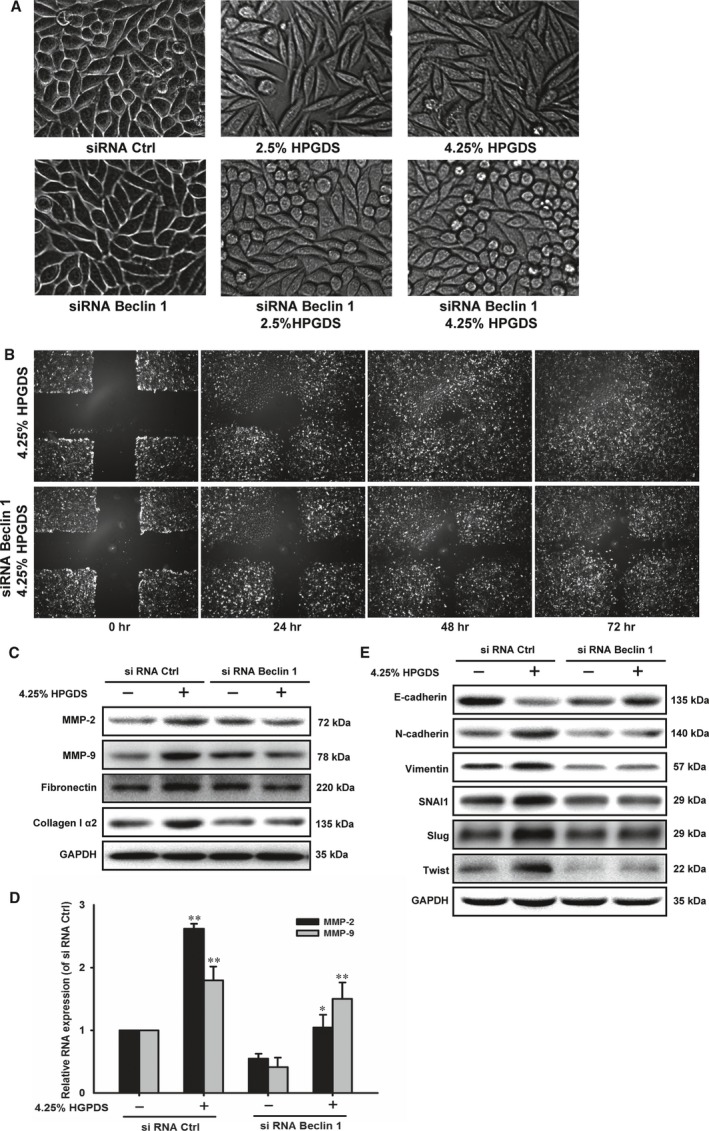
Autophagy inhibition reduced EMT and fibrosis in HPMCs. (**A**) Morphologic changes of cultured HPMCs (Magnification 100×). Human peritoneal mesothelial cells (HPMCs) transfected with siRNA Ctrl or siRNA Beclin 1 were treated with 2.5% high‐glucose peritoneal dialysis solution (HGPDS), 4.25% HGPDS for 24 hrs. Normal cells grew like characteristic cobblestone. Cells treated with HGPDS showed fibroblast morphology. Transfection with siRNA Beclin 1 attenuated such changes of cell morphology. (**B**) HPMCs were plated in 6‐well plates, and a cross scratch was wounded with a sterile micropipette tip. Cells transfected with siRNA Beclin 1 or vehicle were treated with 4.25% HGPDS for 24, 48, and 72 hrs after wounding. Then the migrating cells in denuded zone were assessed using an inverted microscope. Image magnification: 10×. (**C**) Western blotting analysis shows the effect of 4.25% HPGDS treatment of cells transfected with siRNA Beclin 1 or siRNA Ctrl on the protein levels of MMP‐2, MMP‐9, Fibronectin and Collagen Iα. (**D**) Effect of 4.25% HPGDS treatment of cells transfected with siRNA Beclin 1 or siRNA Ctrl on the RNA levels of MMP‐2 and MMP‐9. Melt curve analysis was employed at the end of each PCR to confirm the specificity of the PCR product. Data were analysed according to the comparative Ct method, and target genes expression of MMP‐2 and MMP‐9 was normalized to GAPDH expression levels in each sample. (**E**) Western blotting analysis shows the effect of 4.25% HPGDS treatment of cells transfected with siRNA Beclin 1 or siRNA Ctrl on the protein levels of epithelial‐mesenchymal transition markers. GAPDH was used as loading control. **P < 0.01, *P < 0.05 versus untreated control group

To confirm the contribution of autophagy in the HGPDS‐induced EMT and fibrosis, we further study the inhibition of autophagy with siRNA Beclin 1 on HPMCs. Cells treated with 2.5% HGPDS, 4.25% HGPDS for 24 hrs. EMT in cells was characterized by morphological changes from an epithelial shape to an elongated shape. Cells in control group grew like characteristic cobblestone. Notably, cells treated with HGPDS showed fibroblast morphology. Transfection with siRNA Beclin 1 weakened autophagy and attenuated the above morphologic changes of cultured HPMCs (Fig. 5A). In our present study, the motility ability of HPMCs was measured by wound‐healing assays. Cells transfected with siRNA Beclin 1 or vehicle were treated with 4.25% HGPDS for 24, 48 and 72 hrs after wounding. The migrating cells in denuded zone induced by 4.25% HGPDS were reduced by autophagy inhibition with siRNA Beclin 1 (Fig. 5B).

Additionally, we detected that EMT and fibrosis markers in HPMCs. Figure 5C firstly illustrated that the protein levels of MMP‐2, MMP‐9, fibronectin and collagen I α2 in HPMCs treated by 4.25% HGPDS were significantly inhibited by siRNA Beclin 1 followed by 4.25% HGPDS treatment. Figure 5D illustrated that the RNA levels of MMP‐2, MMP‐9, in HPMCs treated by 4.25% HGPDS were significantly inhibited by siRNA Beclin 1 followed by 4.25% HGPDS treatment. Figure 5E then revealed that EMT markers (N‐cadherin, vimentin, SNAI1, Slug and Twist) in HPMCs treated by 4.25% HGPDS were significantly inhibited by siRNA Beclin 1 followed by 4.25% HGPDS treatment. Thus, autophagy inhibition reduced EMT and fibrosis in HPMCs.

### Autophagy inhibition reduced apoptosis in HPMCs

**Figure 6:**
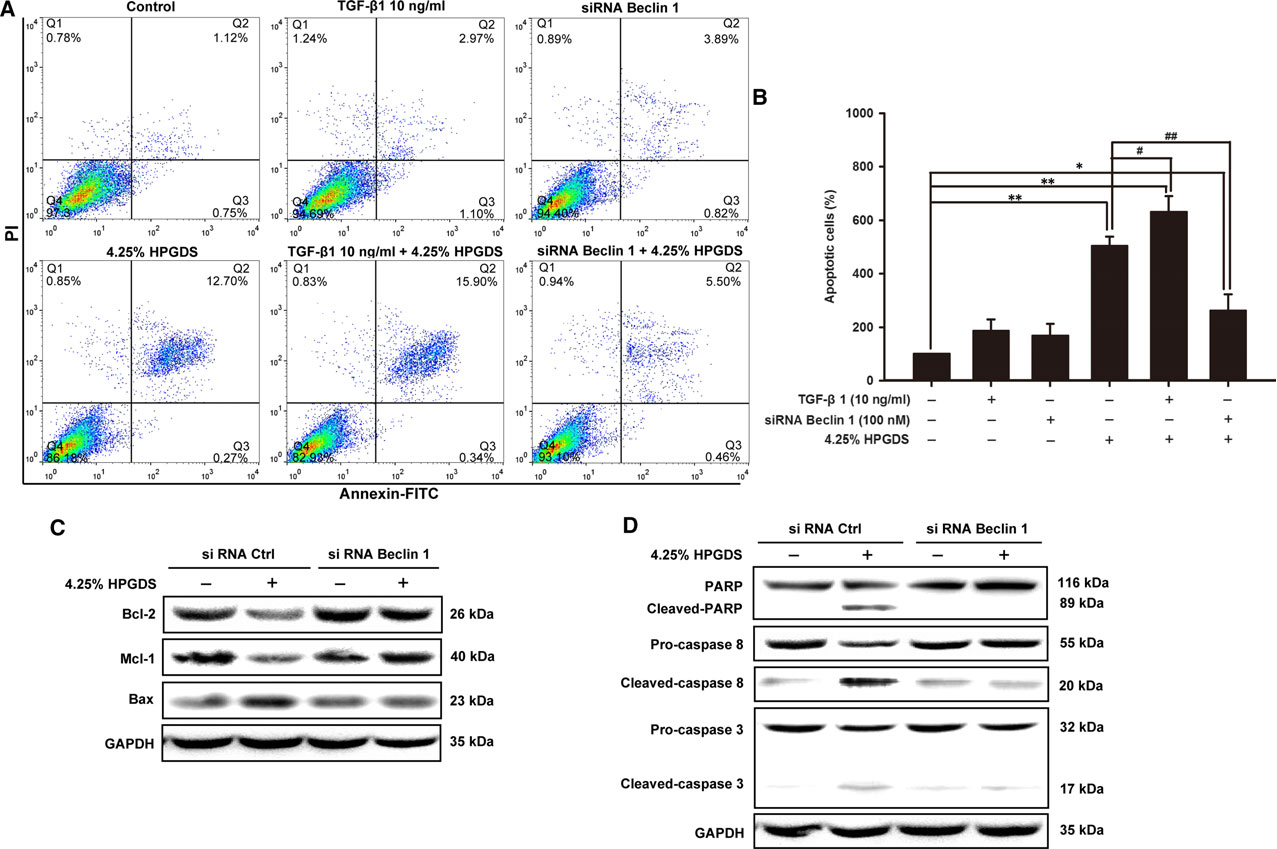
Autophagy inhibition rescued apoptosis in human peritoneal mesothelial cells. (**A** and **B**) Human peritoneal mesothelial cells pre‐treated with TGF‐β1 (10 ng/ml) or transfected with siRNA Beclin1 were treated with 4.25% high‐glucose peritoneal dialysis solution (HGPDS) or vehicle for 24 hrs. The sum of early and late apoptotic cells ratio (%) was quantitated by flowcytometer analysis of Annexin V/PI. Results from three independent experiments are shown as means ± S.D. Results from three independent experiments are shown as means ± S.D. **P < 0.01, *P < 0.05 *versus* untreated control group. ^##^ *P* < 0.01, ^#^ *P* < 0.05 *versus* 4.25% HGPDS‐treated group. (**C** and **D**) Human peritoneal mesothelial cells transfected with siRNA Beclin1 were treated with 4.25% HGPDS or vehicle for 24 hrs. The apoptosis‐related protein such as Bax, Bcl‐2, Mcl‐1, PARP, caspase‐3 and caspase‐8 was assessed by Western blotting assay. GAPDH was used as loading control.

In addition, we performed Annexin V/PI assay to explore whether the autophagy inhibition on apoptosis in HPMCs. The results in Figure 6A and B showed that 4.25% HGPDS could induce apoptosis with the apoptotic cell ratio of 12.21 ± 0.84%. While the apoptosis induction of 4.25% HPGDS was increased by TGF‐β1 (10 ng/ml) (from 12.21 ± 0.84% to 15.28 ± 1.45%). In contrast, the apoptotic cells induced by 4.25% HGPDS were reduced by autophagy inhibition with siRNA Beclin 1 (from 12.21 ± 0.84% to 6.34 ± 1.48%).

We then evaluated the expression of apoptotic protein Bax, the anti‐apoptotic proteins Bcl‐2 and Mcl‐1, caspases 3, caspases 8 and PARP using Western blotting. The results showed that the increased Bax expression and decreased expression Bcl‐2 and Mcl‐1 in HPMCs treated by 4.25% HGPDS were significantly reversed by siRNA Beclin 1 followed by 4.25% HGPDS treatment (Fig. 6C). In addition, compared with 4.25% HGPDS treatment, siRNA Beclin 1 followed by 4.25% HGPDS treatment reduced the cleavage of caspase3, caspase 8 and PARP (Fig. 6D). These results demonstrated that the intrinsic apoptotic pathway involved in 4.25% HGPDS‐induced apoptosis was reduced by autophagy inhibition.

## Discussion

Peritoneal dialysis in patients with ESRF is an important means of renal replacement therapy. Long‐term PD is accompanied by functional and histopathological alterations in the peritoneum. In the long process of PD, HGPDS will aggravate the PF, leading to decreased effectiveness of PD and ultrafiltration failure. PF (or sclerosis) is the most consistent peritoneal structural alterations observed in the PM tissues of patients who undergo long‐term PD therapy 24. The pathogenesis of PF is characterized by the decrease of mesothelial cells, angiogenesis and progressive submesothelial thickening with an increasing presence of myofibroblasts 26. Conventional HGPDS, characterized by possessing high glucose with lactate, glucose degradation products promote pathological fibrosis *via* induction of epithelial‐to‐mesenchymal transition (EMT) process 27. Subsequently, PM undergoes fibrosis and apoptosis, promoting PM morphological and functional failure 28. As reported previously, the human peritoneal mesothelial cell biology contributes to the components and function of PD. HPMCs represent the largest population of resident cells with primary function to supply an adhesive‐free and protective layer against pathogen and damage 29. Transforming growth factor‐beta 1 (TGF‐β1) is considered as the master molecule of fibrogenic cytokines from the injured environment in EMT and PF progression 30.

Effective interventions on the prevention and treatment of PF are rather deficient now. Although the pathogenesis involved in PF is multifactorial, the mechanism of ultrafiltration failure and PD termination is still not fully elucidated in PD. Herein, we demonstrated that HGPDS increased TGF‐β1 production, activated TGF‐β1/Smad2/3 signalling and induced autophagy, fibrosis and apoptosis hallmarks in HPMCs. HGPDS‐induced apoptosis and fibrosis were associated with a persistent induction of Beclin 1‐dependent autophagy. Furthermore, among the different events controlling the pathological process of PM injury, EMT of HPMCs plays a dominant role in fibrosis and in subsequent functional deterioration of the PM 11. Given the central role of EMT and apoptosis in the progression of peritoneal injury, our data provide evidence that autophagy inhibition reduced EMT and apoptosis in HPMCs for the role of autophagy as a modifiable target to protect the PM *via* consistent exposure of HGPDS in the PM.

Previous studies show that EMT and apoptosis of peritoneal mesothelial cells are the initial and reversible mechanisms of PF in PD. However, the detailed regulation mechanism of HGPDS on changes in the microenvironment and PF remains unclear. In this study, firstly, we found the phenomenon in the human PM tissues from patient with PD that the elevated TGF‐β1 expression was consistent with the hallmarks of autophagy and fibrosis (Fig. 1). Transmission electron microscopy analysis of PMs tissues of patients with PD revealed increasing structures of autophagy, apoptosis and fibrosis. There was the characteristic morphology of cell substructure such as autophagosome, autophagolysosmes, typical collagen fibre and apoptotic empty vacuoles under transmission electron microscope (Fig. 2). These findings highlighted a crosstalk among autophagy, fibrosis and apoptosis in human PM tissues from patient with long‐term PD. Thus, we proposed hypothesis what was the interaction among them. Then our results illustrated that HGPDS increased TGF‐β1 production, activated TGF‐β1/Smad2/3 signalling and induced autophagy, fibrosis and apoptosis hallmarks in HPMCs. Indeed, the overexpression of TGF‐β Receptor I and TGF‐β Receptor II was accompanied with hallmarks of autophagy, fibrosis and ECM production in HPMCs, which was the context ignition of TGF‐β1/Smad2/3 signalling activation (Fig. 3).

Autophagy is the tightly catabolic mechanism of cellular components, such as the cytoplasm, organelles and functional protein. The dynamic progress contains the formation of special double‐membrane structure known as the autophagosome, fusion with lysosome to form autolysosome, where the components inside will be digested by the lysosomal 31, 32. As autophagy is a double‐edged sword, it can maintain the cell homeostasis for the supplementary nutrition from the bulk degradation; in the other hand, it can communicate with the cell proliferation for the promotion to apoptosis as type II programmed cell death, or the initiation of fibrogenic changes in morphology and behaviour of epithelial cell such as EMT. Transmission electron microscopy analysis of HPMCs treated with 4.25% HGPDS was applied to gain insight into the cell substructure morphological changes. As a gold standard to detect autophagosome, images from TEM revealed accumulation of autophagy‐related structures and apoptosis empty vacuole in 4.25% HGPDS‐treated cells compared to control cells. Based on the illustration in TEM images, we further demonstrate that Beclin 1 is involved in the autophagy induction, indicating that HGPDS‐induced Beclin 1‐dependent autophagy in HPMCs (Fig. 4).

Recent developments reveal a potential and crucial role for autophagy pathway during human PM induced by HGPDS. Thus, we further study the inhibition of autophagy with siRNA Beclin 1 on HPMCs to confirm the contribution of autophagy in the HGPDS‐induced EMT, fibrosis and apoptosis. Our results demonstrate that autophagy inhibition reduced EMT, fibrosis and apoptosis in HPMCs. These studies are expected to open a new avenue in the understanding of PF, which may guide us to explore the compounds targeting autophagy and achieve the therapeutic improvement of PD.

## Conflict of interests

The authors declare that they have no conflict of interests.
